# Vision-Based Grasping Method for Prosthetic Hands via Geometry and Symmetry Axis Recognition

**DOI:** 10.3390/biomimetics10040242

**Published:** 2025-04-15

**Authors:** Yi Zhang, Yanwei Xie, Qian Zhao, Xiaolei Xu, Hua Deng, Nianen Yi

**Affiliations:** 1 State Key Laboratory of Precision Manufacturing for Extreme Service Performance, Central South University, Changsha 410083, China; zhangyicsu@csu.edu.cn (Y.Z.); breeze_xieyw@163.com (Y.X.); gxdx_zhaoqian@163.com (Q.Z.); xuxiaolei@csu.edu.cn (X.X.);; 2 College of Mechanical & Electrical Engineering, Central South University, Changsha 410083, China

**Keywords:** prosthetic hand, grasp mode, visual recognition, object features, adaptive grasp

## Abstract

This paper proposes a grasping method for prosthetic hands based on object geometry and symmetry axis. The method utilizes computer vision to extract the geometric shape, spatial position, and symmetry axis of target objects and selects appropriate grasping modes and postures through the extracted features. First, grasping patterns are classified based on the analysis of hand-grasping movements. A mapping relationship between object geometry and grasp patterns is established. Then, target object images are captured using binocular depth cameras, and the YOLO algorithm is employed for object detection. The SIFT algorithm is applied to extract the object’s symmetry axis, thereby determining the optimal grasp point and initial hand posture. An experimental platform is built based on a seven-degree-of-freedom (7-DoF) robotic arm and a multi-mode prosthetic hand to conduct grasping experiments on objects with different characteristics. Experimental results demonstrate that the proposed method achieves high accuracy and real-time performance in recognizing object geometric features. The system can automatically match appropriate grasp modes according to object features, improving grasp stability and success rate.

## 1. Introduction

When grasping an object, humans first acquire its geometric configuration, spatial position, and orientation through visual perception. Then, grasping modes and postures are adaptively adjusted based on observed object characteristics, ultimately selecting the optimal grasping mode and posture to achieve stable object acquisition [1]. Several studies have statistically classified human hand-grasping actions. Abbasi proposed a grasp classification method based on force domains by analyzing the force distribution patterns of different grasping techniques [2]. Feix introduced a classification system for static single-hand grasps, categorizing hand grasps into 33 types through four dimensions: opposition type, virtual finger configuration, force/precision classification, and thumb position [3]. Liu integrated static grasping and dynamic hand manipulation and demonstrated the significant influence of object shape, size, and relative position on grasping behavior through behavior experiments [4]. Building upon previous classifications, Stival collected electromyography (EMG) and kinematic data during grasping tasks to develop motion-based and myoelectric classification methods for hand movements [5]. In summary, the human hand exhibits diverse grasping capabilities, allowing it to adapt to various objects.

Regarding adapting to different objects, the soft grippers have better adaptability than the rigid-link robot hands. They can safely grasp completely different objects, such as shapes and materials. Yoon’s entirely passive robot finger was designed by combining the Hart link and parallel mechanism, which effectively imitates the grasping strategy of the human hand when facing environmental constraints [6]. By introducing spring elements and a continuum structure, Sun achieved a grasp similar to the distributed deformation of the human hand. The gripper can flexibly adapt to the uncertainty and complexity of the shape of the object [7]. However, to make the prosthetic hand have a similar shape and grasp movement effect to the human hand, most prosthetic hands are mainly designed by connecting rod mechanisms.

With advancements in assistive technologies, prosthetic devices have significantly improved the quality of life for amputees [8]. In particular, for individuals with upper limb loss, modern prosthetic hands restore essential activities of daily living, requiring enhanced dexterity and multimodal grasping capabilities in anthropomorphic robotic hands [9,10]. To enable prosthetic hands to mimic human grasping abilities, various dexterous hands have been developed. These devices typically offer multiple grasping modes, allowing the selection of appropriate actions based on object characteristics [11]. Currently, available clinical prosthetic control strategies include manual operation, electroencephalography (EEG) decoding, electromyography (EMG) decoding, and visual recognition [12,13,14]. Manual operation requires repetitive user intervention before each grasp attempt, resulting in low autonomy, poor efficiency, and operational inconvenience for amputees. EEG-based control remains technically immature due to challenges such as significant inter-subject variability, susceptibility to signal interference, and limited recognition accuracy. EMG decoding interprets motion intent from residual limb muscle signals to actuate prosthetic devices. However, it requires extensive user training and suffers from latency and signal instability issues. In contrast, vision-based recognition has demonstrated technical maturity and real-time responsiveness, enabling rapid feature extraction while eliminating the need for patient-specific training due to its inherent adaptability [15].

Current research demonstrates the successful integration of visual recognition into prosthetic grasping systems. DeGol was the first to integrate a camera into the wrist of the prosthetic hand, using visual recognition to determine the grasping mode [16]. Cui proposed a method for upper limb motion intention recognition that fused pose and visual data. The grasping intention was determined by obtaining the upper limb pose angle and the target object category [17]. Cognolato introduced a multimodal prosthetic control method based on natural eye-hand coordination through eye tracking and computer vision, proving that visual information can significantly improve the accuracy of grip classification [18]. Pneg used two cameras to perceive the environment of the prosthetic hand and combine tactile and auditory information to grasp the object [19]. Sato installed multiple cameras on the prosthetic hand and implemented visual servo control, demonstrating that the equipment can assist the prosthetic hand in accurately grasping the target object [20]. Weiner integrated a miniature camera inside the palm of the prosthetic hand, displaying the real-time image information collected by the camera on a display on the back of the hand [21]. Shi proposed a global vision-based approach to prosthetic hand control, enabling the prosthetic hand to select a matching grasp pattern based on the target object [22]. Nevertheless, existing studies predominantly focus on single-mode grasping tasks, while research on multimodal grasping remains comparatively underdeveloped.

Contemporary prosthetic hands primarily rely on multi-finger synergies to achieve distinct grasping modes. However, their fixed inter-finger coupling ratios and limited actuation patterns constrain autonomous adaptation to object characteristics. While vision-guided prosthetic research has demonstrated object acquisition capabilities through visual recognition, critical parameters such as geometric properties, spatial pose estimation, and optimal grasp configuration selection remain insufficiently integrated into control architectures. These limitations result in prolonged operation latency and reduced success rates. Consequently, developing an object feature-driven grasping methodology that adaptively selects grasping modes, points, and postures holds significant potential for enhancing the success rate, operational efficiency, and functional versatility in prosthetic applications.

This study proposes an object feature-driven grasping method for multimodal prosthetic hands. The experimental platform integrates a multi-mode prosthetic hand with an anthropomorphic robotic arm, where feature recognition and depth perception enable automatic selection of optimal grasping mode and posture. This system achieves efficient object recognition and adaptive grasping through real-time feature extraction of object geometry and spatial relationships.

## 2. Division of Grasping Mode

**Figure 1:**
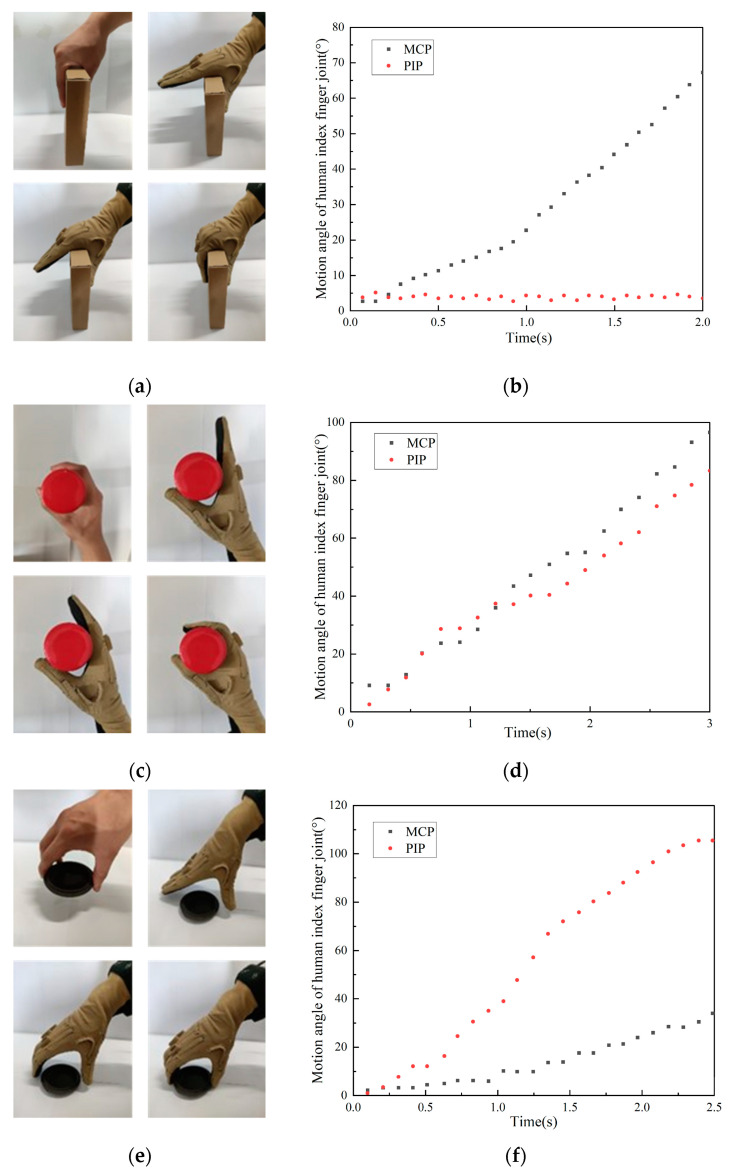
Manual grasp of different objects and joint angle data. (**a**) Grasp the paper box with a thickness of 35 mm. (**b**) Joint angle data when grasping the paper box. (**c**) Grasp the water cup with a diameter of 50 mm. (**d**) Joint angle data when grasping the water cup. (**e**) Grasp the plate with a diameter of 80 mm. (**f**) Joint angle data when grasping the plate.

During human grasping, appropriate modes are selected according to object geometry and size. For different objects, the coupling relationship between each phalangeal joint varies during grasping. As shown in Figure 1, angle data of the metacarpophalangeal joint (MCP) and proximal middle phalangeal joint (PIP) of the index finger of the human hand are collected during the grasping of different objects using the CyberGlove II. As shown in Figure 1b,d,f, the coupling ratio of joints varies when grasping different objects. It can be concluded from Figure 1f that the velocity of the PIP changes with the angle of the MCP. The coupling state between the fingers of the human hand can be divided into three distinct types: uncoupled, fixed-coupling, and variable-coupling. Correspondingly, grasping modes are classified as (1) Uncoupled adaptive mode, (2) Fixed-coupling adaptive mode, and (3) Variable-coupling adaptive mode. Each mode exhibits unique kinematic characteristics when combined with thumb opposition.

**Figure 2:**
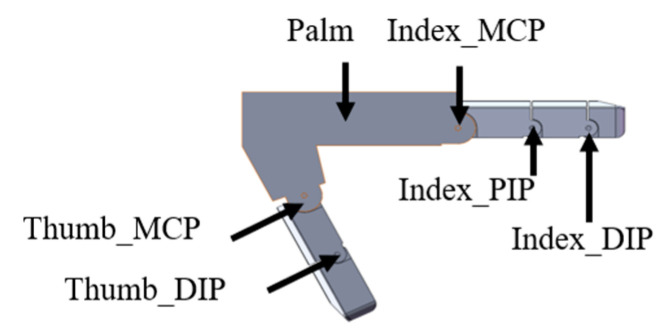
Schematic diagram of prosthetic hand grasping simulation system.

Based on the coupling ratios among the phalangeal joints of human hands when grasping objects, we established a simulation system for grasping prosthetic hands, as shown in Figure 2. It consists of an index finger and a thumb. Each phalanx is designed based on the average finger joint length of a healthy adult. The index finger can adopt the three distinct grasping modes by changing the coupling ratio between the phalangeal joints. The thumb mainly plays an auxiliary role in grasping, using fixed-coupling adaptive mode.

**Figure 3:**
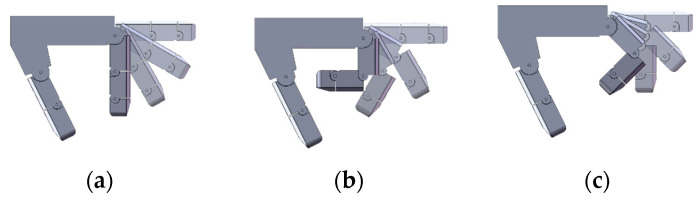
Schematic diagram of grasping trajectory. (**a**) Uncoupled adaptive mode. (**b**) Fixed-coupling adaptive mode. (**c**) Variable-coupling adaptive mode.

The distal phalanx of the index finger is merged with the middle phalanx due to length constraints. Doing so does not affect grasp performance [23]. Meanwhile, the two-phalanx design meets lightweight requirements. Therefore, the middle and distal phalanges are merged into a unified distal phalanx (index_dip). The pre-contact grasping trajectories of the three grasping modes are illustrated in Figure 3. Through data analysis and fitting in Figure 1b,d,f, the velocity relationship between proximal phalanges (index_mcp) and distal phalanges of each model can be obtained as follows:

(1) $$(v_{mcp},v_{pip})=\left\{\begin{array}{l} (v,\alpha v),\;\text{MCP-}\;\&\;\text{DIP-}\;\\ (0,\beta v),\;\mathrm{MCP}+\&\;\text{DIP-}\\ (0,0),\;\mathrm{DIP}+ \end{array}\right.$$

(2) $$(\alpha ,\beta )=\left\{\begin{array}{l} (0,1),\;\mathrm{Uncoupling}\;\mathrm{adaptive}\;\mathrm{mode}\\ (1,1),\;\text{Fixed-copuling}\;\mathrm{adaptive}\;\mathrm{mode}\\ (f(\theta_{mcp}),f(\theta_{mcp})),\;\text{Variable-coupling}\;\mathrm{adaptive}\;\mathrm{mode} \end{array}\right.$$

(3) $$f(\theta_{mcp})=\left\{\begin{array}{l} 3,\;\theta_{mcp}<15^{\circ }\\ 2,\;15^{\circ }<\theta_{mcp}<90^{\circ } \end{array}\right.$$

α and β represent the coupling ratios. The MCP+ and DIP+ indicate that the corresponding knuckle is in contact with the object. The MCP- and DIP- indicate that the corresponding joints are not in contact with the object. The value of $f\left(\theta_{mcp}\right)$ can be derived from the data in Figure 1f. According to Equations (1)–(3), the motion relationship of the knuckles in each mode can be obtained.

**Figure 4:**
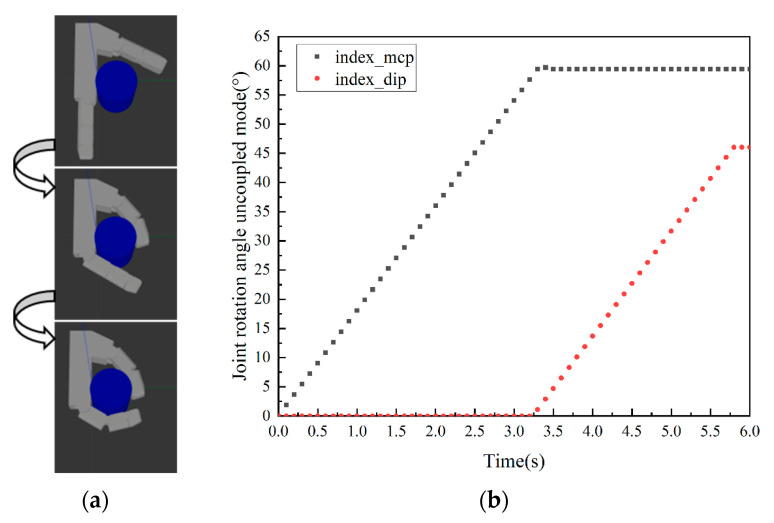
Grasping cylinder in uncoupled mode. (**a**) Grasping process diagram. (**b**) Rotation angle data of the index finger’s proximal and distal phalanges.

**Figure 5:**
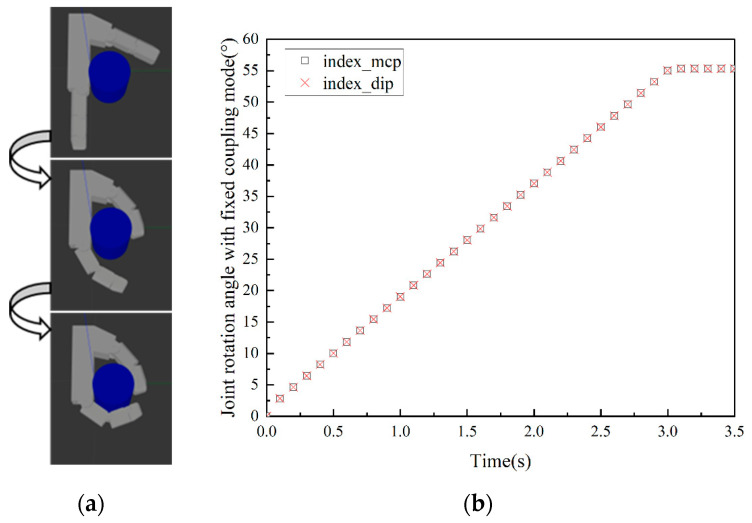
Grasping cylinder in fixed-coupling adaptive mode. (**a**) Grasping process diagram. (**b**) Rotation angle data of the index finger’s proximal and distal phalanges.

**Figure 6:**
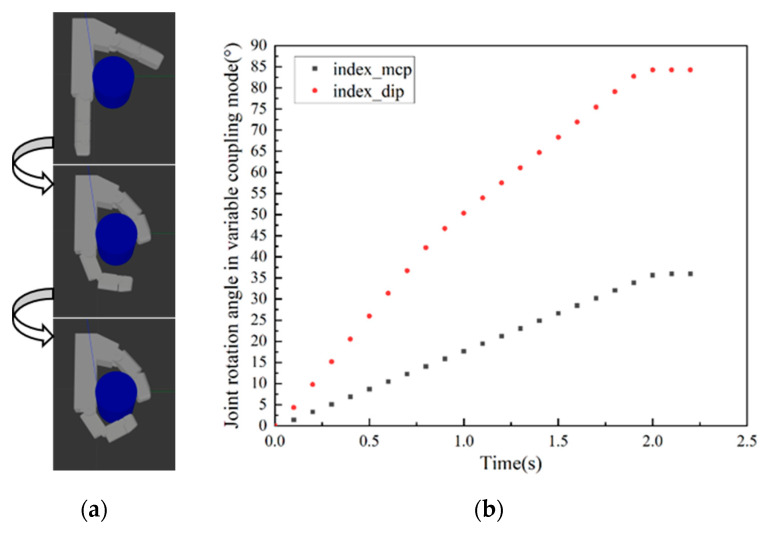
Grasping cylinder in variable-coupling mode. (**a**) Grasping process diagram. (**b**) Rotation angle data of the index finger’s proximal and distal phalanges.

According to the established grasping simulation system, the grasping ability of the prosthetic hand in three modes can be tested. Grasping performance is judged by grasping time and contact area to determine the best grasping object type for each mode. A cylinder with a diameter of 60 mm and a height of 100 mm is first grasped. Based on practical experience, it is suitable for enveloping the object. The simulation results are shown in Figure 4, Figure 5 and Figure 6, and all three grasping modes can grasp the cylinder. But in the uncoupled mode, as shown in Figure 4b, the distal phalanx only begins to move when the proximal phalanx touches the cylinder. Therefore, it takes more time to complete the grasp than the other two modes. It can be seen from Figure 5 and Figure 6 that the variable-coupling adaptive mode takes less grasping time than the fixed-coupling adaptive mode. However, variable-coupling grasping results in a reduced contact area between the fingers and the target object. Under identical actuation torques, the fixed-coupling mode yields greater fingertip grasping force.

**Figure 7:**
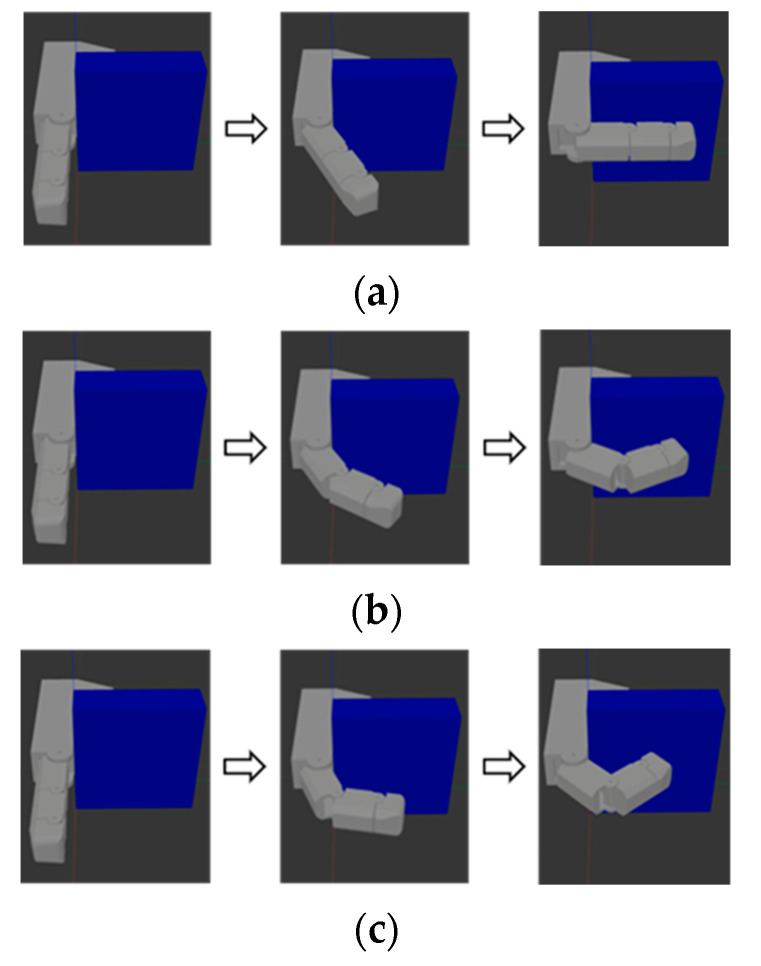
Schematic diagram of grasping a tablet object. (**a**) Uncoupled adaptive mode. (**b**) Fixed-coupling adaptive mode. (**c**) Variable-coupling adaptive mode.

The second object to be grasped is a tablet-shaped object measuring 100 mm in length, 30 mm in width, and 100 mm in height. In the uncoupled adaptive mode, owing to its motion characteristics, the prosthetic hand’s index finger can fully contact the object when an appropriate grasp point is selected, as shown in Figure 7a. In contrast, owing to the coupling characteristics of the fixed-coupling and variable-coupling adaptive modes, only the distal phalanx contacts the object, as shown in Figure 7b,c. Under identical driving torques, the uncoupled adaptive mode achieves a greater grasp force.

**Figure 8:**
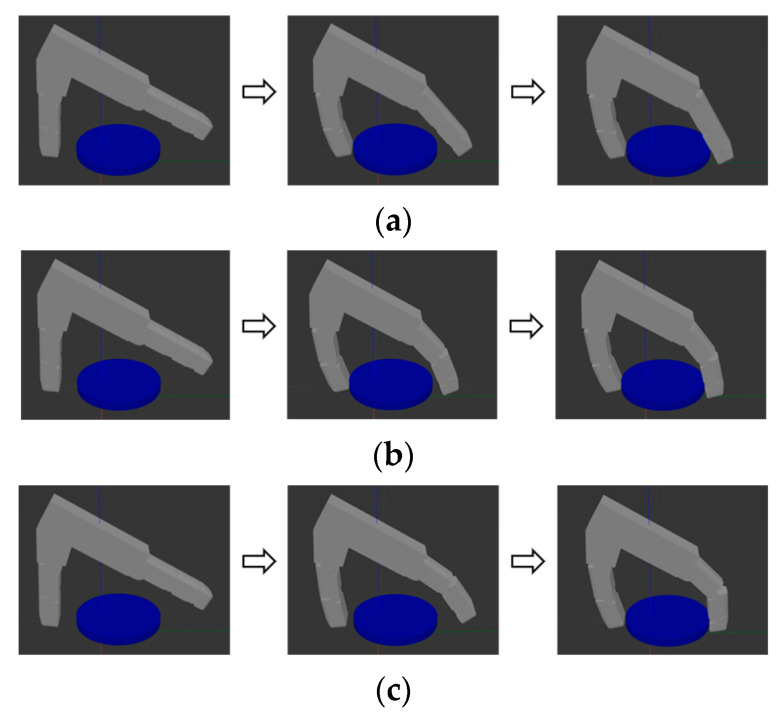
Schematic diagram of grasping a flat object. (**a**) Uncoupled adaptive mode. (**b**) Fixed-coupling adaptive mode. (**c**) Variable-coupling adaptive mode.

The third object to be grasped is a flat object with a radial size of 50 mm and an axial size of 10 mm, positioned on a table. Its relatively large cross-sectional area makes it suitable for prosthetic hands to perform pinching or claw-like grasps from above. As shown in Figure 8a, in the uncoupled adaptive mode, the distal phalanx initiates movement only after the proximal phalanx contacts the object, which makes it unsuitable for grasping objects with large cross-sectional areas. In the fixed-coupling adaptive mode, the distal phalanx’s contact angle constraints hinder the effective transfer of grasping force to the object’s surface, as depicted in Figure 8b. In the variable-coupling mode, the distal phalanx of the index finger achieves near-vertical contact with the object, as shown in Figure 8c. This allows the driving force to be applied more effectively to the object’s surface, thereby enhancing grasp stability. Driving forces can be applied to the surface more effectively, thus improving grasp stability.

Analysis of the grasping characteristics of the three prosthetic hand modes reveals that each mode is best suited to a specific type of object. Common objects are classified into five categories based on their geometric shapes: cylinder, globe, cuboid, tablet, and ellipsoid. Table 1 presents the mapping between an object’s geometric shape and the corresponding finger movement pattern.

## 3. Feature Based Grasping Method

**Figure 9:**
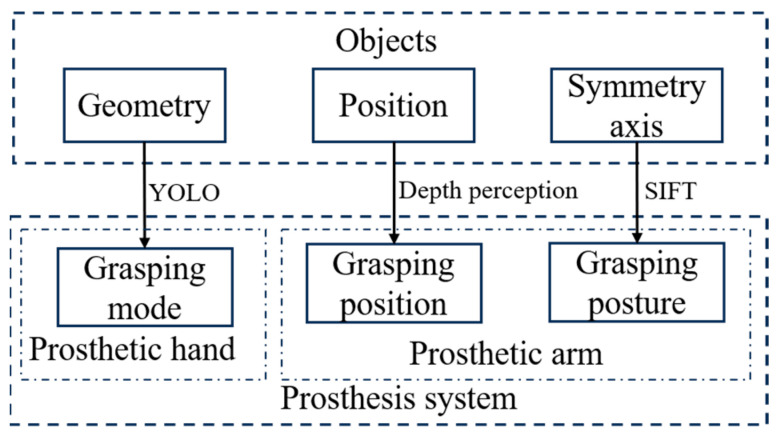
Grasping method structure diagram.

The proposed grasping method requires the prosthetic hand to select the corresponding grasping mode based on the geometric shape of the grasped object. The visual recognition algorithm is introduced in the grasping process to recognize the geometric shape features of the object. The prosthetic hand can choose the grasping mode based on the recognized geometric shape. At the same time, the grasping points and grasping posture corresponding to the prosthetic hand can be reasonably selected by combining the characteristics of the object. The overall structure of the grasp method is shown in Figure 9.

### 3.1. Object Geometry Recognition

#### 3.1.1. Dataset Establishment

**Figure 10:**
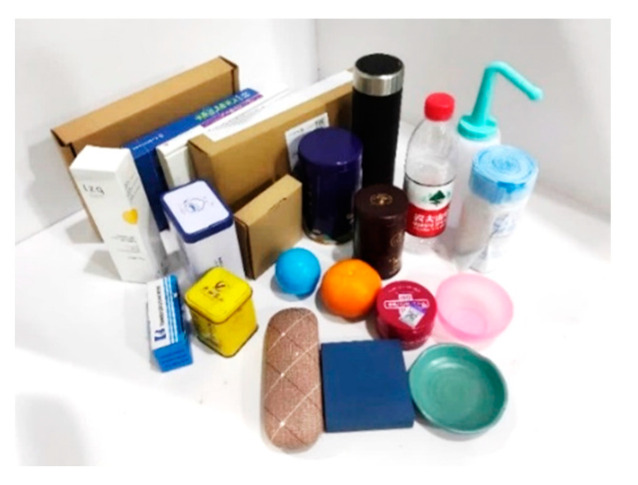
Twenty-two objects are selected to build the dataset.

**Figure 11:**
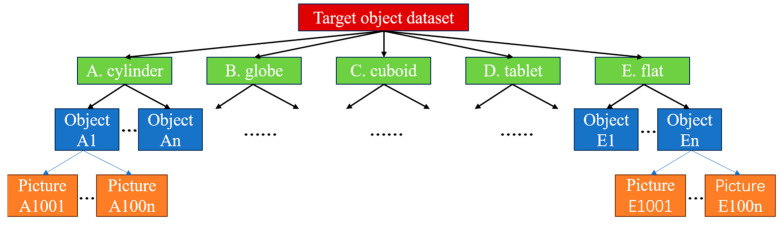
Structure of the target object dataset.

In daily prosthetic hand applications, 22 objects (as shown in Figure 10) are selected to establish a dataset and train the model. A binocular depth camera is mounted on the base via a bracket. The objects are placed at distances between 20 cm and 60 cm from the camera, and over 1000 photos of the 22 objects are collected. Based on the structure shown in Figure 11, a dataset is established using a class-based photo structure. According to the geometric shape of objects, the dataset is divided into five categories.

#### 3.1.2. Training of Neural Network Models

Using the established target object dataset, we train a neural network model through object detection algorithms. In subsequent applications, the geometric shape of the object can be quickly and accurately identified. Considering that the objects grasped by prosthetic hands are mostly common in daily life, there is a certain requirement for detection speed. In practical applications, multiple detection targets may appear in the field of view, so we choose to use the YOLO algorithm for object detection [24]. And use the YOLOv3-tiny small network as the detection algorithm for target objects.

**Figure 12:**
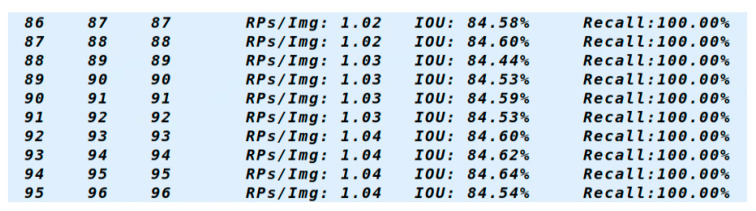
Model test results.

The entire dataset is divided into training and testing sets in a 9:1 ratio. This division can effectively evaluate the accuracy and generalization ability of the model, making the model performance stable and effective. After training, the model is tested, and the results are presented in Figure 12. A total of 96 images were selected for the test, and all the ground truth could be correctly detected. It can be concluded that the IOU reached 84% and the recall reached 100%, indicating that the model has good performance.

**Figure 13:**
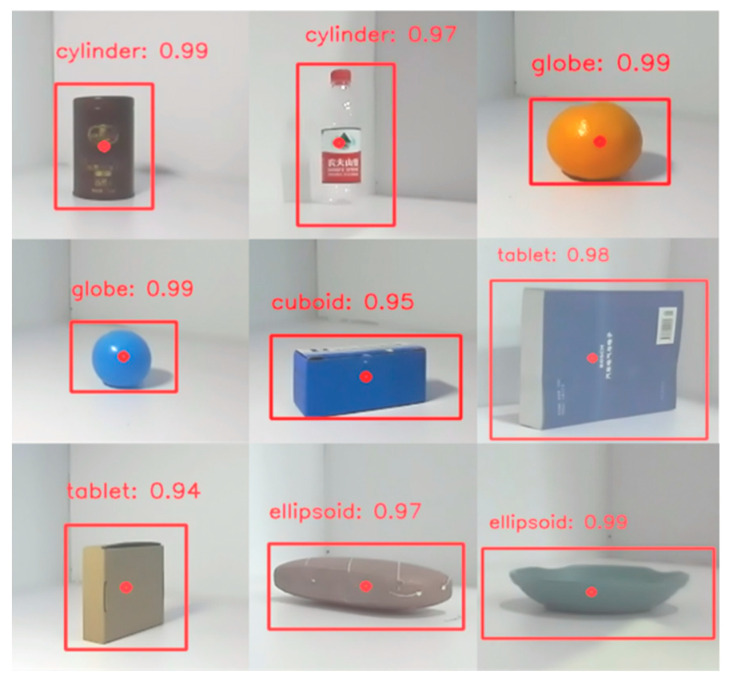
Results and confidence levels of model recognition of object geometry.

To evaluate the model’s ability to recognize object geometry, several objects with different geometric shapes are selected for testing. Figure 13 displays the model’s geometric shape recognition results along with the corresponding confidence levels for each object. The model not only correctly identified the geometric shapes of all eight objects but also achieved confidence levels of 0.94 or higher for each. In addition, tests show that the model can recognize the shape of objects in under one second. These results demonstrate that the model can identify object geometry quickly, accurately, and reliably.

### 3.2. Acquisition of Object Position Information

**Figure 14:**
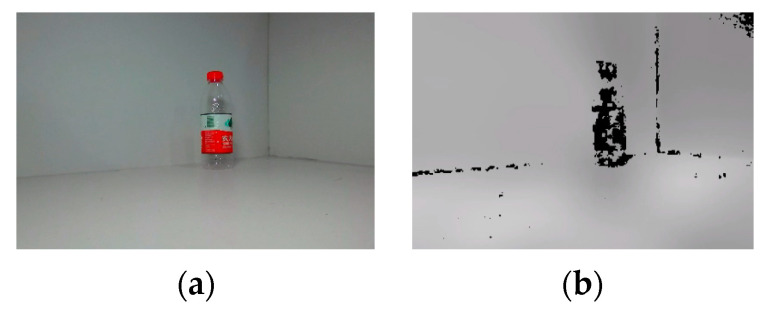
RGB image and depth map of a water bottle. (**a**) RGB image. (**b**) Depth map.

The example object is a water bottle. The RGB image and depth map captured by the binocular depth camera for the object are shown in Figure 14. After extracting the object’s depth values in the camera coordinate system, the depth map and RGB image are registered for 3D reconstruction. After registration, a 3D point cloud of the object is generated.

**Figure 15:**
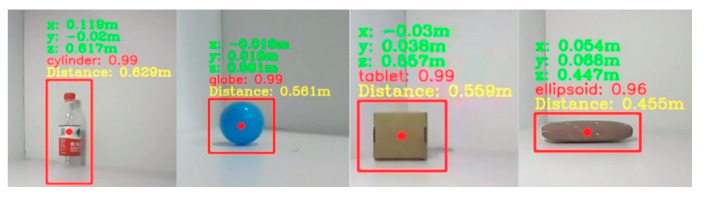
Object position coordinate information and geometric shape recognition results.

The position information of the target object can be extracted from the 3D point cloud generated by the depth camera. As shown in Figure 15, four objects are selected and tested to evaluate the accuracy of position extraction. Figure 15 displays the recognition results, showing the three-dimensional coordinates of each object in the left camera coordinate system. The Euclidean distance between the object’s center and the left camera coordinate system origin was calculated. After manual measurement, the position coordinate error of the object extracted from the 3D point cloud is within 0.5 cm, indicating that the positional information can be accurately obtained. In addition, the model’s object geometry recognition results, along with the associated confidence levels, are presented.

### 3.3. Object Symmetry Axis Detection

In this section, the symmetry axis of the object is extracted using a symmetry axis detection algorithm based on the Scale Invariant Feature Transform (SIFT). This allows the prosthetic hand to select an appropriate grasping posture based on the object’s axis of symmetry to grasp the object [25]. We take a paper box as the test object and describe the implementation process of each step of the object symmetry axis detection algorithm.

**Figure 16:**
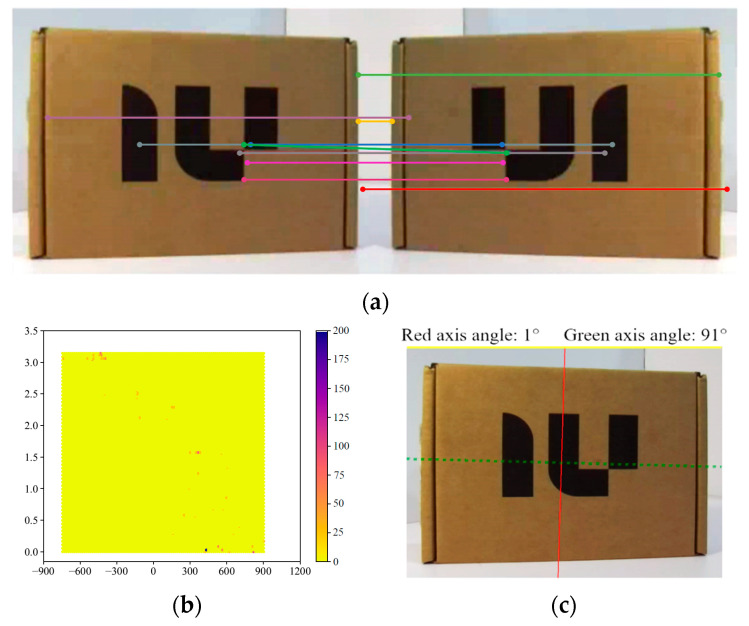
Symmetry axis detection process. (**a**) The top ten feature point pairs and the endpoints of each line represent a pair of feature points. (**b**) Hexbin diagram. (**c**) Symmetry axis detection result of a paper box.

(1) Extract the image regions from each object prediction box and use these image regions as input images for the symmetry axis detection algorithm.
(2) Mirror the carton image. And convert both the original and mirrored images into array matrices, a collection of row and column index points. The SIFT algorithm is applied to detect key points in the image, which are points that can be reliably detected and matched under varying scales and rotation conditions.
(3) The Brute-Force Matcher (BFMatcher) is used to match the symmetric key points between the original image and the specular reflection image, and a set of matched feature point pairs is generated. According to the distance between each pair of feature points, a smaller distance indicates better symmetry matching. Figure 16a shows the top 10 pairs of feature points.
(4) The Hexagonal Binning (Hexbin) diagram is used to display all the count results, as shown in Figure 16b. The region with the highest count corresponds (the darkest color), according to the mirror line that draws the image of the object. It’s the symmetry axis of the object.

After the above steps, the symmetry axis of the carton can be detected, as shown in Figure 16c. Most objects have a single axis of symmetry. To better determine the prosthetic hand’s posture when grasping objects, the symmetry axis detected by the algorithm is defined as the principal symmetry axis. Based on geometric constraints, an auxiliary symmetry axis perpendicular to the principal symmetry axis is drawn at the center of the object image. In Figure 16c, the red axis is the symmetry axis detected by the algorithm, and the green axis is the symmetry axis drawn according to the geometric relationship. The angle between the axis and the image border is also displayed, clockwise is positive.

**Figure 17:**
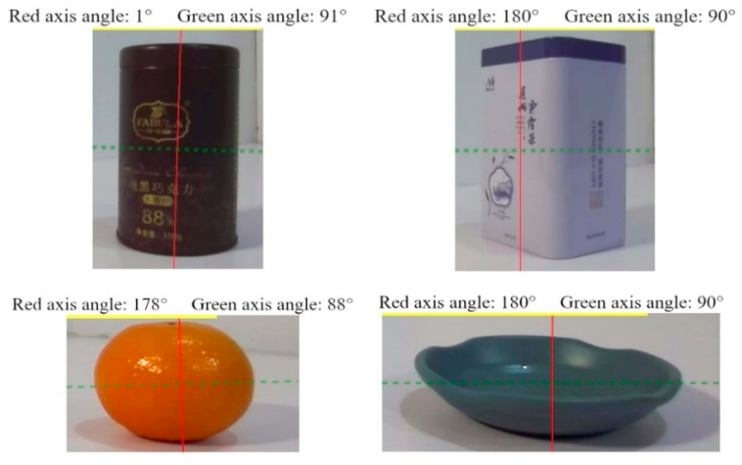
Symmetrical axis detection results of several objects.

To verify the effectiveness of the symmetry axis detection algorithm, several different objects are tested. As shown in Figure 17, the symmetry axis of these objects can be accurately identified.

#### 3.3.1. Determination of Grasp Posture

According to the length of the symmetry axis, it can be divided into long axis and short axis. Based on the characteristics of the symmetry axis and three grasping modes, the grasping posture of the prosthetic hand can be determined.

When the recognized object is a tablet-shaped object, such as a book, cardboard box, or dining plate. According to Table 1, the uncoupled adaptive mode should be used to grasp such objects. At the beginning, the palm surface of the prosthetic hand should be perpendicular to the short axis of the object, and the fingers should grasp along the short axis.

When the recognized object is a cylinder, globe, or cuboid, such as water bottles, thermos cups, apples, and tea cans. According to Table 1, the fixed-coupling adaptive mode should be used to grasp such objects. Combining the characteristics of objects and the grasping habits of human hands. At the initial stage, the palm surface of the prosthetic hand should be parallel to the short axis of the object, and the thumb and index finger should grasp along the short axis direction of the object.

When the object is ellipsoid, such as eyeglass cases, cosmetic cans, and headphone cases. According to Table 1, the variable-coupling adaptive mode claw grasp should be used for such objects. Based on the characteristics of a large cross-sectional area, short longitudinal dimensions, and prosthetic claw grasp of the object. The prosthetic hand should be located directly above the object and grasped along the short axis of the object at an angle of about 45° between the palm surface and the long axis of the object.

#### 3.3.2. Determination of Grasp Points

**Figure 18:**
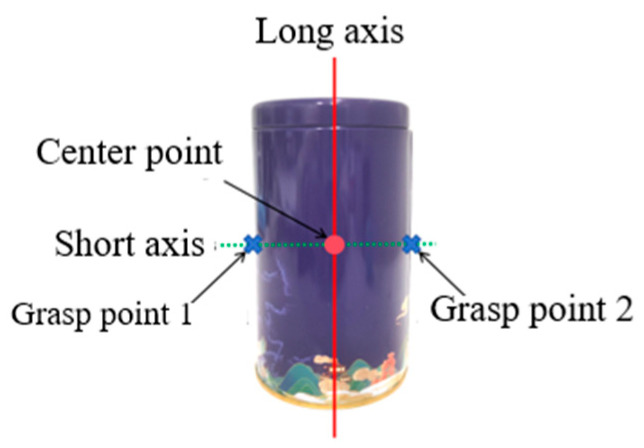
The center point and grasp point of the cylindrical metal can.

The selection of grasping points significantly affects the success rate and stability of the grasp. For example, when grasping a cylindrical metal can, as shown in Figure 18, its geometric shape can be identified as a cylinder. According to Table 1, the appropriate grasping mode is determined to be the fixed-coupling adaptive mode. The center coordinates of the object can be obtained from the three-dimensional point cloud. Using the previously mentioned method to determine the grasping posture, it is clear that the cylindrical metal can should be grasped along its short axis.

The outline size of an object can be extracted from its three-dimensional point cloud. The grasping point is determined by translating the center coordinates of the object in the camera coordinate system. In this example, the prosthetic hand uses a fixed-coupling adaptive mode to grasp the metal can by enveloping it along the short axis. To do this, the width of the metal can along the short axis must first be obtained. Then, by translating and transforming the center coordinates along the short axis, two grasp points are generated: grasp point 1 and grasp point 2. During the actual grasping process, the appropriate grasp point should be selected to differentiate between the left and right hands.

Similarly, for the uncoupled adaptive mode. Assuming the object is placed in front of the camera, and the main feature plane of the object faces the camera (the front of a flat paper box facing the camera). Enable the camera to accurately capture the symmetry axis of the object. From the grasping posture in Figure 7a, it can be seen that the grasping point of the object in this mode should mainly consider the length dimension along the short axis direction. For the variable-coupling adaptive mode, it can be seen from the grasping posture in Figure 8c that the height dimension of the object in the short-axis direction is the main consideration factor.

## 4. Experiment

In the previous content, a grasping method based on object features is proposed, and the grasping mode, grasping posture, and grasping point of the prosthetic hand are determined. Next, experiments will be conducted to verify the grasping method.

### 4.1. Introduction to the Experimental Platform

**Figure 19:**
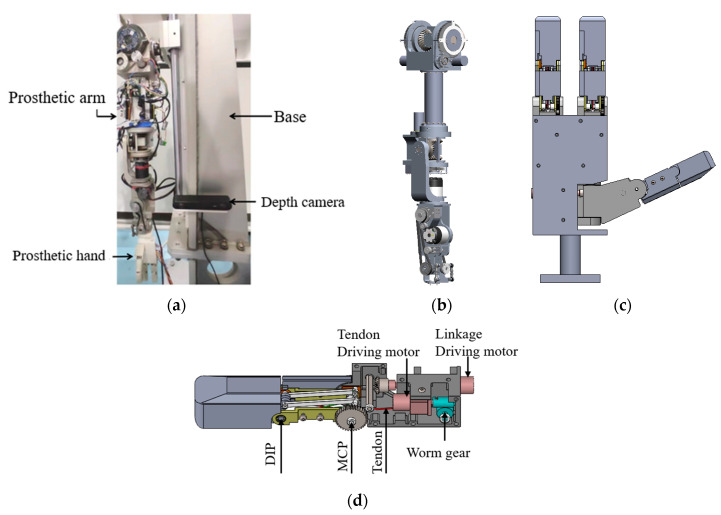
Grasp experimental platform based on object features. (**a**) Experimental platform. (**b**) 3D model of prosthetic arm. (**c**) 3D model of a multi-mode prosthetic hand. (**d**) The finger’s 3D internal structure.

The grasping experimental platform incorporates an anthropomorphic 7-DOF prosthetic arm, a multi-mode prosthetic hand, and a binocular depth camera, as shown in Figure 19a. The prosthetic arm is a 7-DoF upper limb prosthetic experimental platform developed in the laboratory, and its main structure is similar to that of a human arm, as shown in Figure 19b. It consists of the shoulder joint, upper arm, elbow joint, forearm, wrist joint, and other parts, with 2-DoF at the shoulder joint and the wrist joint, respectively, and 3-DoF at the elbow joint. For its control strategies, dynamic modeling, trajectory planning, and trajectory tracking implementation refer to Reference [26]. The prosthetic hand design employs a hybrid transmission mechanism integrating linkages and tendon-driven systems. Micro-motors dynamically regulate tendon lengths and tension states to modulate interphalangeal coupling ratios. It can implement the three grasping modes proposed in the previous article. The model is shown in Figure 19c, and its grasp ability and control method can be referred to in reference [27]. Additionally, the internal three-dimensional structure of the finger is shown in Figure 19d. The depth camera is ZED2 from STEREOLABS, which has a resolution of 2K and a frame rate of up to 100 FPS.

### 4.2. Grasp Experiment

Typical objects suitable for three grasping modes are selected for grasping experiments. They are a paper box with a thickness of 58 mm, a cylindrical metal can with a diameter of 80 mm, and a plastic bowl with a size of 88 × 55 mm.

**Figure 20:**
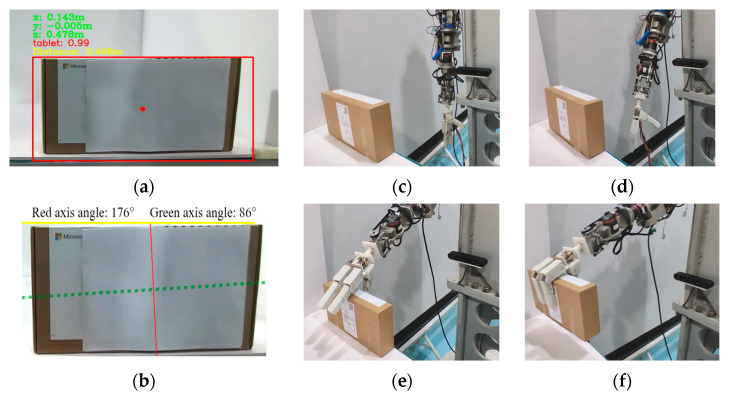
Action flow when grasping a cardboard box with a thickness of 58 mm. (**a**) Geometric shape and position recognition results and confidence level of the cardboard box. (**b**) Symmetrical axis detection results of the cardboard box. (**c**) Initialization of prosthetic arm and prosthetic hand. (**d**) Movement of the prosthetic arm to the designated position based on hand-eye calibration results. (**e**) The prosthetic arm moves to the grasp point. (**f**) According to the mapping relationship of grasping modes, select the uncoupled mode to grasp the cardboard box and lift it.

For the cardboard box, as shown in Figure 20a, its geometric shape can be visually recognized as a tablet. According to Table 1, the uncoupled grasp mode should be used. The center coordinates of the cardboard box in the camera coordinate system are (0.143, −0.005, 0.478), with units in meters. The distance between the center of the cardboard box and the origin of the camera coordinate system is 0.499 m. The symmetry axis detection result is shown in Figure 20b, with the long axis (dashed line) as the auxiliary symmetry axis and the short axis (solid line) as the main symmetry axis. According to the grasping posture determination method, the prosthetic hand should have the palm surface perpendicular to the short axis of the paper box, and the fingers should grasp along the short axis. From the three-dimensional point cloud of the cardboard box, the outline dimensions along the short axis of the carton are extracted. The upper grasp point of the cardboard box is used as the grasp point for the prosthetic hand to grasp. Based on the above content, the target pose of the prosthetic arm end in the camera coordinate system can be obtained. As shown in Figure 20c–f, the end pose of the prosthetic arm in the base coordinate system can be derived according to the hand-eye calibration relationship. When the prosthetic arm moves to the target position, the prosthetic hand completes the grasp of the cardboard box in the uncoupled adaptive mode and lifts it.

**Figure 21:**
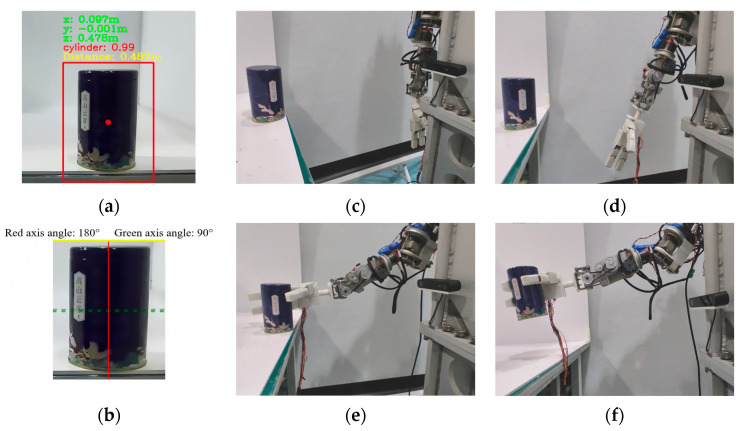
Action flow when grasping a cylindrical metal can with a diameter of 80 mm. (**a**) Geometric shape and position recognition results and confidence level of the metal can. (**b**) Symmetrical axis detection results of the metal can. (**c**) Initialization of prosthetic arm and prosthetic hand. (**d**) Movement of the prosthetic arm to the designated position based on hand-eye calibration results. (**e**) The prosthetic arm moves to the grasp point. (**f**) According to the mapping relationship of grasping modes, select the uncoupled mode to grasp the metal can and lift it.

For the cylindrical metal can, as shown in Figure 21a,b, based on visual recognition and symmetry axis detection, it can be inferred that its geometric shape is cylindrical. The center coordinates of the metal can in the camera coordinate system are (0.097, −0.001, 0.478), with units in meters. The distance from the origin of the camera coordinate system is 0.487 m. The long axis (solid line) is the main symmetry axis, and the short axis (dashed line) is the auxiliary symmetry axis. The right grasp point on the short axis should be selected, and the prosthetic palm should be parallel to the long axis of the metal can in the fixed-coupling adaptive mode, with the thumb and finger grasping along the short axis. As shown in Figure 21c–f, the end pose of the prosthetic arm in the base coordinate system was derived using the hand-eye calibration relationship. When the prosthetic arm moves to the target position, the prosthetic hand completes the grasp of the metal can in the fixed-coupling adaptive mode and lifts it.

**Figure 22:**
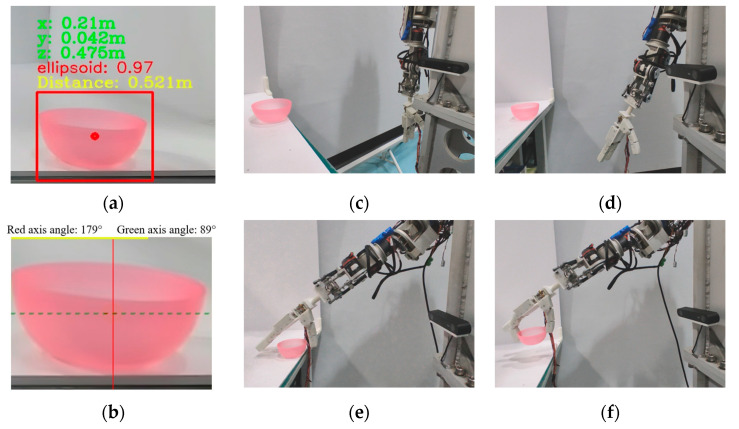
Action flow when grasping a plastic bowl with a size of 88 × 55 mm. (**a**) Geometric shape and position recognition results and confidence level of the plastic bowl. (**b**) Symmetrical axis detection results of the plastic bowl. (**c**) Initialization of prosthetic arm and prosthetic hand. (**d**) Movement of the prosthetic arm to the designated position based on hand-eye calibration results. (**e**) The prosthetic arm moves to the grasp point. (**f**) According to the mapping relationship of grasping modes, select the uncoupled mode to grasp the plastic bowl and lift it.

For the plastic bowl, as shown in Figure 22a,b, based on visual recognition and symmetry axis detection, it can be inferred that its geometric shape is ellipsoid. The center coordinates of the plastic bowl in the camera coordinate system are (0.21, 0.042, 0.475), with units in meters. The distance from the origin of the camera coordinate system is 0.521 m. The long axis (dashed line) is the auxiliary symmetry axis, and the short axis (solid line) is the main symmetry axis. The upper grasp point should be selected. The prosthetic hand should be located directly above the object. At an angle of about 45° between the palm surface and the long axis of the plastic bowl, the variable-coupling adaptive mode should be used to grasp along the short axis direction. As shown in Figure 22c–f, the end pose of the prosthetic arm in the base coordinate system was derived using the hand-eye calibration relationship. When the prosthetic arm moves to the target position, the prosthetic hand completes the grasp of the plastic bowl in the variable-coupling adaptive mode and lifts it.

**Figure 23:**
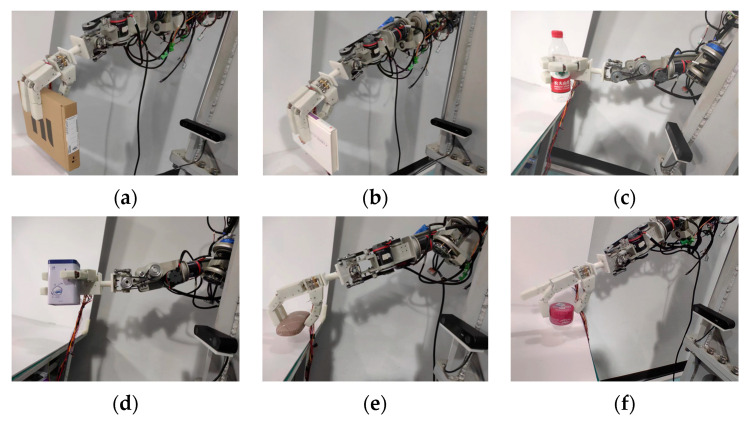
Experimental results of grasping various objects. (**a**,**b**) Uncoupled adaptive mode grasping tablet objects. (**c**,**d**) Fixed-coupling adaptive mode grasping cylindrical and rectangular objects. (**e**,**f**) Variable-coupling adaptive mode grasping flat objects.

In addition, several other objects are also selected for the grasping experiment, and the results are shown in Figure 23. The experimental results indicate that the grasping mode and posture can be correctly selected during the grasping process of different objects. And successfully completed the grasp experiment.

## 5. Discussion

The proposed vision-based grasping method demonstrates promising performance in recognizing object geometry and selecting adaptive grasping modes for prosthetic hands. Experimental results indicate that the system achieves high accuracy in object feature extraction and completes grasping tasks for diverse objects. By combining geometric shape classification with symmetry axis detection, the method enables precise grasp point and posture selection. This dual-feature approach mimics human grasping behavior. Moreover, this method uses lightweight algorithms to ensure fast feature extraction. This is essential for the grasping process, which requires low latency. Feature recognition speed, in line with the actual requirements of control. Compared to EMG-based or EEG-based prosthetic control strategies, the vision-driven approach eliminates the need for user training and mitigates signal instability issues. Since the core principle of the proposed method is the different coupling ratios of the finger joint and object feature recognition, commercial dexterous hands can better adapt to this grasping method after the introduction of vision. Whether the soft gripper is used in the proposed grasping method should be analyzed according to the structure of the finger. If they can realize different coupling ratios of finger joints, the grasping method proposed in this paper can also be realized.

Since this method relies only on visual input, problems such as poor ambient field of view, such as occlusion, may reduce detection accuracy. The lack of haptic feedback results in reduced robustness to sliding or unexpected disturbances.

## 6. Conclusions

In conclusion, based on the characteristics of the human hand grasping different objects, this paper proposes a grasping method based on object geometry and symmetry axis. Firstly, the mapping relationship between grasping modes and geometric features of objects is established through the simulation of several grasping modes of prosthetic hands. Secondly, a prosthetic hand grasping mode and posture automatic selection method based on object geometry and position information is proposed. Finally, an experimental platform is built to validate the proposed method. The experimental results show that this method can accurately identify the geometric shapes of common objects in daily life and achieve reliable grasping through reasonable grasping patterns.

In our future work, the following problems need to be solved. That is the real-time adjustment of the grasping force of objects with different characteristics during the grasping process. Fortunately, we proposed a compliant control method for prosthetic hands based on object stiffness estimation and based on muscle stiffness in our previous work [28,29]. In the future, we will try to combine these two methods into the grasping method proposed in this paper to achieve a more stable grasping.

## Figures and Tables

**Table 1:** Mapping relationship between object geometry and finger movement patterns.

Geometric Shape of Object	Prosthetic Hand Finger Movement Mode
cylinder	Fixed-coupling adaptive mode
globe	Fixed-coupling adaptive mode
cuboid	Fixed-coupling adaptive mode
tablet	Uncoupled adaptive mode
ellipsoid	Variable-coupling adaptive mode

## Data Availability

The data and code of the current study can be obtained from the corresponding author upon reasonable request.
